# Access to HIV prevention services among gender based violence survivors in Tanzania

**Published:** 2012-12-25

**Authors:** Beati Mboya, Florence Temu, Bayoum Awadhi, Zubeda Ngware, Elly Ndyetabura, Gloria Kiondo, Janneth Maridadi

**Affiliations:** 1 African Medical and Research Foundation (AMREF), Dar es Salaam, Tanzania; 2 Tanzania Field Epidemiology and Laboratory Training Program (FILTEP), Dar es Salaam, Tanzania; 3 United Nations Development Program (UNDP), Dar es Salaam, Tanzania; 4 Ministry of Health and Social Welfare (MOHSW), Dar es Salaam, Tanzania

**Keywords:** Gender based violence, HIV, people living with HIV, survivor, health services, access

## Abstract

**Introduction:**

Currently, Tanzania's HIV prevalence is 5.7%. Gender inequality and Gender Based Violence (GBV) are among factors fuelling the spread of HIV in Tanzania. This study was conducted to assess universal access to HIV prevention services among GBV survivors in Iringa and Dar-es-Salaam where HIV prevalence is as high as 14.7% and 9% respectively compared to a national average of 5.7%.

**Methods:**

In 2010, a mixed methods study using triangulation model was conducted in Iringa and Dar-es-Salaam regions to represent rural and urban settings respectively. Questionnaires were administered to 283 randomly selected survivors and 37 health providers while 28 in-depth interviews and 16 focus group discussions were conducted among various stakeholders. Quantitative data was analyzed in SPSS by comparing descriptive statistics while qualitative data was analyzed using thematic framework approach.

**Results:**

Counseling and testing was the most common type of HIV prevention services received by GBV survivors (29%). Obstacles for HIV prevention among GBV survivors included: stigma, male dominance culture and fear of marital separation. Bribery in service delivery points, lack of confidentiality, inadequate GBV knowledge among health providers, and fear of being involved in legal matters were mentioned to be additional obstacles to service accessibility by survivors. Reported consequences of GBV included: psychological problems, physical trauma, chronic illness, HIV infection.

**Conclusion:**

GBV related stigma and cultural norms are obstacles to HIV services accessibility. Initiation of friendly health services, integration of GBV into HIV services and community based interventions addressing GBV related stigma and cultural norms are recommended.

## Introduction

Gender based violence occurs in different setting and various forms that include trafficking and sexual violence [1, 2]. Sexual Violence involves “any sexual act, attempt to obtain a sexual act, unwanted sexual comments or advances, or acts to traffic a person's sexuality, using coercion, threats of harm or physical force, by any person regardless of relationship to the survivor, in any setting, including but not limited to home and work” [1]. GBV is caused and perpetuated by deeply-rooted factors including poverty, social acceptance, tolerance, weak actions against perpetrators and traditional gender norms with prevailing male dominance [3]. Exposure to GBV among women increases the chances of high risk sexual practices such as forced sex and non-use of contraception such as condoms. This results in poor reproductive health outcomes such as unintended pregnancies, unsafe abortion, and HIV/STIs transmission through traumatic abrasions and high risk of HIV infection as perpetrators are likely to force unprotected sex. Voluntary migration and trafficking of young men and women as a form of GBV have recently been highlighted as widespread in Tanzania [2, 4] with serious sexual and reproductive health risks particularly to affected women and girls. Migration of young people from rural to urban areas come into play, voluntarily or through human-domestic trafficking, and again heightening exposure to GBV and risk of HIV infection. Parents are involved in trafficking young girls, all driven by poverty, lack of opportunities and employment in rural areas, misguided belief of a better life in urban setting and the need for financial support [5, 6].

There is growing evidence that GBV can increase the risk of HIV/AIDS as well as outcome of the disease. Several studies undertaken in the U.S. and South Africa indicate a positive relationship between HIV/AIDS incidence and domestic violence [7, 8]. A multi-centric study in Latin America and the Caribbean profiling domestic violence showed a positive association between STIs and domestic violence. Tanzania is among the Sub-Saharan African countries with a high HIV prevalence and there is documentation in 2001, supporting that HIV-positive women were over 2.5 times more likely to have experienced violence at the hands of their current partner than other women [8, 9]. Despite the association between GBV and HIV, stigma and fear among women survivors hinder disclosure of events, limiting access to GBV related services [10].

This study assessed access for HIV prevention, treatment and support among GBV survivors (women, girls and children) in targeted communities in Iringa and Dar-es-Salaam where HIV prevalence is as high as 14.7% and 9% respectively compared to a national average of 5.7% [11]. Specifically the study determined the socio demographic factors of GBV survivors of domestic trafficking, sexual exploitation and abuse in relation to HIV infection; types and causes of GBV among survivors and assessed availability and survivor's access to HIV prevention services. While acknowledging the comprehensive definition and survivors of GBV as being beyond women and children, in this study the concept of GBV included domestic trafficking, sexual abuse and exploitation.

## Methods

This mixed methods study using triangulation model was conducted in 2010 in Iringa and Dar-es-Salaam regions in Tanzania [12]. It involved concurrent collection of data, and integrates both quantitative and qualitative data in the results, interpretation and conclusion. The two regions were selected because of high HIV prevalence above national figure of 5.7% (16% and 9% respectively). Iringa is a source while Dar-es-Salaam is major destination for trafficked girls. Different stakeholder views and perceptions on GBV awareness and related factors as well as GBV survivors’ accessibility to relevant services were collected using semi-structured interviews and focus group discussions. Health care providers and GBV survivors’ views on GBV and health services accessibility were collected using standardized questionnaires. A two stage sampling procedure was used. The first stage involved purposive selection of three districts representing urban, peri-urban and rural setting and random selection of one ward in each district to participate in the study. In each of the participating districts, the following key stakeholders were purposively selected for interview: District Executive Directors, District Medical Officers, Social welfare Officers, Community Development Officers, Ward Executive Directors, health Officers and health facility in charges.

The second stage involved selection of health facilities, safe houses, famous pubs, bars and brothels located in the three districts. Pubs and bars were selected randomly from lists of similar facilities in the study ward in each district while all safe houses and brothels located in the study districts were selected. All GBV survivors found in the selected safe houses, brothels, pubs and bars were invited to participate in the study if they were meeting the eligibility criteria which included suffering from at least one GBV event in a life time, aged 18 – 49 years and willing to participate. Three district hospitals, six randomly selected health centres (2 from each study ward) and two regional hospitals from each region participated in the study. A total of 37 health care providers were randomly selected and invited to participate in the study (3 from each district hospital and health centre and 5 from each regional hospital). Six to eight PLHIV were randomly selected from a PLHIV group randomly selected from a list of PLHIV support groups in each of the study ward. The selected PLHIV members were invited to participate in this study.

In depth interviews were used to generate data from government officials, key opinion leaders, and selected community members. Interviews were conducted under supervision of the authors by trained research assistants who were able to communicate in both Swahili and English. Probing was applied during the interview wherever necessary to enrich the data collected and help in understanding the issues in detail [13]. Focus group discussions were used to collect data from GBV survivors, PLHIV groups, community groups (youth, adult male and female groups) because it is the best method for generating data from several people in a short period of time given the time constrain in accomplishing this study [14]. Focus group discussions were conducted in Swahili and were moderated by trained and native Swahili speaking research assistants. The focus groups were homogeneous in nature taking into account age groups, sex, education and HIV status. There were GBV survivors, health providers, youth males, youth females, adult males, adult females, PLHIV male and PLHIV female groups from each of the two regions. The researcher wanted participation from each participant while avoiding lack of cohesion and side conversations which usually occur in large groups, hence six participants were allocated per group [15, 16]. The focus group discussions were conducted in meeting rooms with privacy. A total of 28 in-depth interviews and 16 focus group discussions were conducted and were adequate to reach saturation point where there was no new emerging issue.

A standardized questionnaire was used to collect quantitative data from GBV survivors and health care providers. The questionnaires were administered to the respondents by trained research assistants that were fluent in both Swahili and English. All research instruments were pretested and adjusted accordingly prior to the data collection exercise. Based on the assumption that our hypothesized anticipated proportion of victims accessing HIV prevention and management services was 30%, our default proportion in the general population was 50%, a sample size of at least 200 gave us a power of 100%. In this study a total of 283 GBV survivors out of the population of GBV survivors in the two regions and 37 health care providers were interviewed.

### Data analysis

**Qualitative data:** Data analysis started right from the beginning of the research period throughout the data collection period. Research assistants were involved in the preliminary data analysis, and they had opportunity to review the arising themes and give their views in relation to the real situation on the ground. After the first few pre-test interviews and FGDs, the guides were reviewed and adjusted. Recorded data was transcribed by research assistants using laptops. The textual data was translated from Swahili to English by two experienced translators and 10% of the scripts were back-translated to Swahili by different translators to cross-check quality and any loss of meaning [17]. MAXQDA software package was used to help manage the data during the analysis especially in organizing and retrieving data. Five steps of the thematic framework approach for qualitative data analysis were followed to analyze the textual data. The researchers repeatedly read all the scripts to get familiarization and deep understanding of the information as well as clarifying areas which were not clear. Thematic framework was drawn from the research objectives and issues arose from the interviews. The data were indexed and grouped into themes and subthemes according to level of generality for easy retrieval, review and further exploration. Charting was conducted; related segments from different transcripts were pulled together and examined to identify concepts. The process of mapping and interpreting of data was shaped by the research objectives and emerging themes [18]. Research assistants participated in selection of themes and quotes presented in findings.

**Quantitative analysis:** Completed questionnaires with quantitative data were cleaned and coded by the research assistants. Cleaned data were entered into SPSS16 database. Quantitative analysis basing on frequencies and proportions was done. Chi-square test was used to compare proportions of variables and a P-value of <0.05 was considered statistically significant.

### Ethical Consideration

Ethical clearance was sought from the National Institute of Medical and Research in Tanzania. Informed consent was sought from participants and permission to record the interviews or focus group discussions was obtained. All participants were assured about privacy, anonymity and confidentiality of the data collected prior to consent.

## Results

### Socio-demographic characteristics of GBV survivors interviewed

Out of 283 GBV survivors 277 were females and 6 were males. The mean age was twenty five (25) years (minimum age=12years, median=22years, mode=16years). Majority of GBV survivors were 15-24 years of age (58.3%). Most of the GBV survivors had primary school education (79.2%). Although majority (58%) was not married, about 87% had one or more children (Table 1).

**Table 1:** Socio-demographic characteristics of the gender based violence survivors interviewed

Socio-demographic characteristics	Frequency	Percent (%)
**Sex**		
Male	6	2.1
Female	277	97.9
**Education**		
None	29	10.2
Primary education	224	79.2
Secondary and above	30	10.6
**Marital Status**		
Not married	172	60.8
Married	52	18.4
Cohabit	9	3.2
Separated/Divorced/Widowed	50	17.6
**Parity**		
0	39	13.8
1	79	27.9
2	57	20.1
3 or more	108	38.2
**Age group (Years)**		
< 15	25	8.8
15 – 24	140	58.3
25 – 34	69	24.4
35 and above	40	17.3

Out of 37 interviewed health care providers, about 70% indicated that women are the most vulnerable groups to gender based violence. Young girls were also perceived to be one of the commonest vulnerable groups; this was perceived by a larger proportion of health care providers in Iringa (58%) than Dar es Salaam (33%).

Qualitative findings suggests that, women particularly young girls experience all type of gender based violence such as being beaten by their husbands, insulted, denied the right to make own decision, raped and sometimes forced to be inherited as per local customs. Male children were reported to be favoured when it comes to education, leaving the girls with low education and more susceptible to GBV.

*“My sister and I managed to pass standard seven exams and we were both accepted to join form one, however my father said that I should proceed with school but my sister must remain at home and wait to get married”* (Community male individual living with HIV).

Widows were commonly mentioned in the in-depth interviews and focus group discussion as a group of individuals likely to experience GBV from relatives as they fight for resources.

*“In our community, women are beaten by their husbands and widows are forced to be inherited without their consent when the husband died, this increases risk of HIV transmission”*(Social Welfare Officer)

### Types and causes of GBV events experienced by survivors

**Figure 1:**
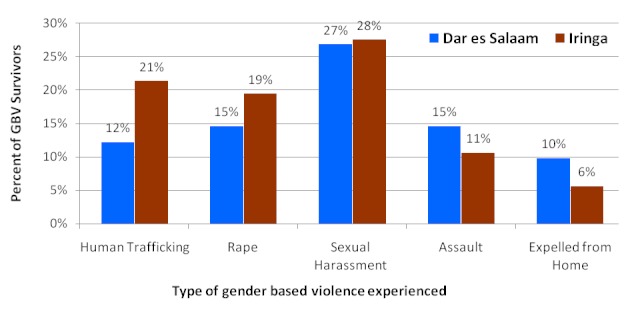
Type of gender based violence (GBV) experienced, by GBV survivors by region (N=283)

**Figure 2:**
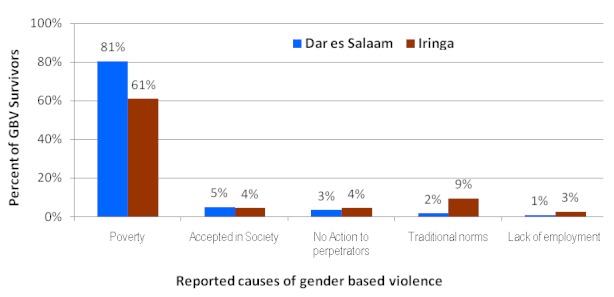
Main causes of gender based violence as reported by survivors by region (N=283)

About two thirds of the survivors interviewed had the view that communities have knowledge on GBV while a third felt that the communities were totally ignorant about gender based violence. Sexual harassment (27%), human trafficking (20) and rape (15%) were the major types of gender-based violence experienced by the interviewed survivors; these were more in Iringa than Dar es Salaam; p < 0.0001 (Figure 1). 78% of health providers interviewed had encountered a case of sexual exploitation or abuse particularly young girls being exploited by older males who are usually their employers (Figure 2). While sexual exploitation was more encountered by health providers in Dar es Salaam, human trafficking cases were encountered more by health providers in Iringa. Qualitative findings showed that relatives living with the survivor were frequently mentioned as perpetrators of GBV.

*“Women are bitten by their husbands”*(Social Welfare Officer).

*“Widows are forced by close relatives to be inherited without their consent when the husband died”* (Community female individual living with HIV).

Among GBV survivors interviewed, friends (32%) and parents (27%) are the commonest perpetrators. Qualitative findings highlighted parents particularly widows as perpetrators of girls trafficking.

*“Widows send their children particularly girls to go to cities and work when they suffer sexual abuse and end up in prostitution”*(Community male individual living with HIV).

Poverty (in this study – ability to meet individual daily basic needs- food, shelter and clothes) was the most mentioned (70%) cause of gender based violence across the study districts followed by traditional norms (male dominance culture) (p=0.001). Poverty was the most common cause of gender based violence among those with primary school or less compared to those with secondary school education or above; p=0.001 (Figure 2).

*“Women are being harassed because of poor income. An able woman with good income cannot be harassed by men”* (Community female individual living with HIV)

Excessive use of alcohol, non-disclosure of GBV event and lack of action to perpetrators were also mentioned as causes of GBV.

*“Excessive drinking of alcohol results in fighting in the family and sometimes marital separation”* (Community male individual)

Focus group discussions showed that HIV influences gender based violence events as a result of existing community stigma and discrimination among individuals testing HIV positive. Orphans are forced to work at early age and are prone to GBV.

*“She was harassed and beaten after disclosing HIV status. Others have decided to keep quiet”* (Community female individual living with HIV)

*“Also diseases like AIDS has given us a lot of problems. Personally I was stigmatized I was not given vegetables”* (Community male individual living with HIV)

**Figure 3:**
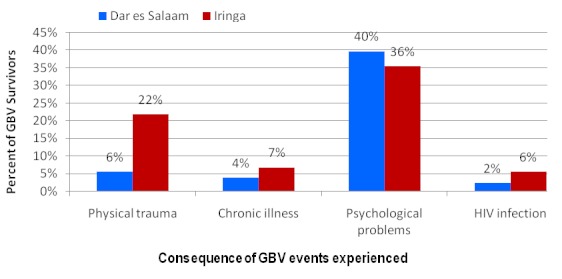
Consequence of gender based violence events as experienced by GBV survivors (N=283)

GBV survivors mentioned psychological problems (27%) as commonest consequences of GBV; others include physical trauma, chronic illness and HIV infection (Figure 3). HIV infection as consequence of GBV event was reported more in Iringa compared to Dar es Salaam (p < 0.0001).

### Availability of HIV prevention services for GBV survivors

**Figure 4:**
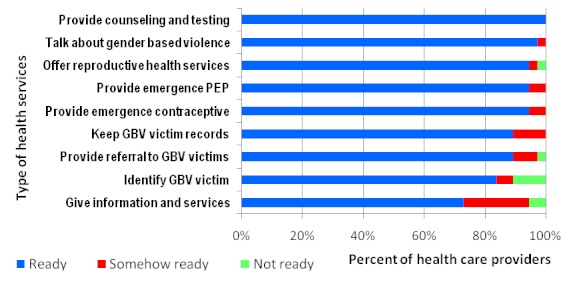
Health providers ability to provide HIV and gender based violence related services (N=37)

**Figure 5:**
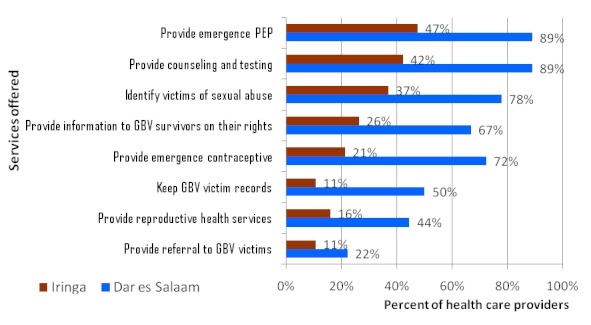
Services offered by health care providers during the past one year (N=37)

The authors were interested with the following services; post exposure prophylaxis, HIV counseling and testing, sexual transmitted illness services and general HIV prevention services. Some of the services mentioned to be available in the community were legal services, health services, and police as well as information services. More than 80 percent of providers agree that they have the right to ask their clients about gender based violence issue and propose for HIV prevention services. All providers interviewed reported to be able to provide counseling and testing. Only 10.8% of providers responded that they are not ready to keep records and information on GBV victims and 5.4% said they are not ready to give referrals to other services for GBV victims (Figure 4 and Figure 5). The availability of ledgers for recording GBV services was only reported by 16.7% of health providers; slightly higher proportion of providers in Dar es Salaam (27.8%) keep ledgers for GBV services compared to health providers in Iringa (5.3%) (P=0.06). During FGD and In-depth interviews, some respondents said some of the doctors do not take detailed history from the GBV victim in order to obtain the so called cause of the problem. Health professionals are not willing to witness in court hence they avoid GBV cases.

*“I had a cousin that was beaten by my uncle, we went to Mwananyamala hospital and went to the first doctor and he said he doesn't want to be involved and taken to court as a witness”*(Female Community member)

### Accessibility of HIV prevention services among GBV survivors

Counseling and testing services was the most common type of HIV services received by GBV survivors (29%) across the study districts (p < 0.0001). Obstacles for HIV prevention, care, treatment and support services among GBV survivors included: stigma and unwillingness to disclose GBV events in the community, fear of marital separation and male dominant culture. Corruption (bribery) in service delivery points, lack of confidentiality, inadequate GBV knowledge and skills among health providers, and fear of being involved in legal matters were mentioned to be additional obstacles to service accessibility by survivors. In qualitative findings, corruption in service delivery points (police and health facility) was mentioned to be obstacle to service accessibility by survivors.

*“When you happen to have money, you get the service first but if you don't have money even if you are sick you can't get the service”* (A female youth community member).

Fear of marital separation among the victims of these gender based violence especially women make them neither to report the event nor to access HIV services.

*“Fear causes them not to look for these services; fear that they will be asked questions to explain. Some do fear the possibility of being seen as dirty”* (A male community member).

Qualitative findings also showed that lack of confidentiality among service providers who are supposed ethically to observe confidentiality also hinder services accessibility by GBV survivors.

*“Because there is no privacy or secret, if I tell him/her about this, it will reach my boss and I will get it ten times more”* (A female PLWHA youth)

## Discussion

Findings from this study reported GBV survivor as a girl child, from poor family, with poor education, unemployed; somebody with few options to enable her to benefit on her human right to basic needs and service accessibility. As shown in other studies, most of the GBV survivors were female from poor families and in particular girl child from poor family [19]. The mean age of GBV survivors interviewed was 24 years and 67.1% were below 25 years. These findings are contrary to those found in other studies where majority of survivors were reported to be below 18 years [20, 21]; for example in a study done in Nepal majority of survivors were below 19 years of age [10].

Although several factors underlying GBV were found, poverty in the family was the major underlying cause of gender based violence particularly trafficking young girls from rural to urban areas to work as bar maids and house girls. Lack of education and unemployment emerged as contributors of GBV. Traditional norms particularly male dominant cultures are not only a cause of GBV but also obstacles to health services accessibility and GBV event reporting. Alcoholism among males is a source of marital disharmony and consequent GBV. Several studies have also shown that alcoholism is associated with high risk behaviors for HIV transmission [22, 23].

The fact that majority of the unmarried GBV survivors had children indicates that they are involved in risky behaviors such as unprotected sex which predispose them to HIV infection. Similarly other studies have shown high rates of unintended pregnancies, symptoms of STI and multiple concurrent partnerships among female survivors of sexual violence [24, 25]. Almost 90% of survivors end up with only primary school education which is not adequate to empower them to get a decent job with enough income to sustain their life, hence they are left with few options and most opt for low paying jobs like house and bar maids which further predispose them to GBV and HIV/AIDS infection. Sexual harassment was the most commonly encountered type of GBV. Similar findings in Tanzania have been published [19]. Although psychological problems were the main consequence of GBV; similar to other studies, HIV infection was reported as a consequence of rape and sexual exploitation [26, 27].

Similar to other studies, relatives living with the survivor particularly biological parents emerged as major perpetrators far beyond human trafficking institutions [28]. Other reasons for girls trafficking from rural to urban areas include; being forced by poverty (failure to meet daily basic needs), search for good life and parents’ willingness to give away there girl children to work in cities. Trafficked girls in cities usually end up in sex work which not only predisposes them to GBV but also to HIV. Although there was good awareness about GBV, reporting and combating GBV problems are suppressed by male dominance and lack of stronger policy and systems to address GBV issues.

As shown in other studies, factors influencing HIV transmission among GBV survivors include poverty, lack of education and school dropouts, culture, and divorce, marital separation, being widow, sexual exploitation and abuse [29]. Therefore GBV issues and HIV transmission risk seem to originate from underlying poverty in the community hence women empowerment programs could be of help.

Services accessibility was also hampered by lack of confidentiality, support and psychological counseling as well as good customer care at health facilities and police stations in case of GBV event. Limited disclosure among GBV survivors have been reported in other studies, for example in a study conducted in Uganda, there was low disclosure of HIV positive women among GBV survivors [30]. Fear of male dominant culture and likelihood of experiencing GBV event among women hinders GBV event disclosure, reporting and HIV service accessibility; these have been reported in other studies [31, 32].

As for other poor general population, bribery, lack of fare and long walking distance were mentioned as obstacles to health services accessibility. Although majority of health providers were willing to provide GBV services, they were not comfortable about keeping GBV data, providing referrals to other services or providing witness at court.

This study is not without limitations. The time for this research was short, however the researchers addressed this by recruiting adequate number of well educated research assistants who were trained and mentored before actual data collection. GBV survivors are usually a difficult group to identify; however the purposive sampling used in this study made it possible; incentives such as snacks were offered during FGDs and interviews. Potential of respondents not opening up during discussions because GBV issues are sensitive was another constraint; however meeting rooms with privacy and confidentiality were used and this enabled participants to open up and contribute a lot in generation of data. In this study GBV survivors were not tested for HIV hence the study was not able to establish HIV status among GBV survivors; this is a potential area for future research.

## Conclusion

Gender based violence related stigma and cultural norms are obstacles to HIV services accessibility. HIV risk behaviors among GBV victims are influenced by poverty, lack of education and school dropouts, male dominance culture, and divorce, marital separation, being widowed, sexual exploitation and unfriendly GBV related services. Initiation of friendly GBV related services, integration of GBV services in HIV prevention and community based interventions addressing GBV related stigma and cultural norms are recommended.
